# Non-invasive Assessment of Coronary Artery Disease: The Role of AI in the Current Status and Future Directions

**DOI:** 10.7759/cureus.78994

**Published:** 2025-02-14

**Authors:** Francis Ezekwueme, Oluwaremilekun Tolu-Akinnawo, Zana Smith, Kayode E Ogunniyi

**Affiliations:** 1 Internal Medicine, University of Pittsburgh Medical Center, Pittsburgh, USA; 2 Internal Medicine, Meharry Medical College, Nashville, USA; 3 Radiology, South East Regional Health Authority, Kingston, JAM; 4 Internal Medicine, University Hospital of North Durham, Durham, GBR

**Keywords:** artificial intelligence (ai), coronary artery disease, deep learning, machine learning, non-invasive

## Abstract

Coronary artery disease (CAD) remains a significant public health concern due to its high morbidity and mortality rates. Early detection and timely evaluation are crucial for improving patient outcomes. While both invasive and non-invasive methods are available for assessing CAD risk, non-invasive approaches minimize the complications associated with invasive procedures. Over the past two decades, advancements in artificial intelligence (AI), particularly machine learning techniques such as deep learning and natural language processing, have revolutionized cardiology. These technologies enhance diagnostic accuracy and clinical efficiency in non-invasive CAD evaluation. However, the broader adoption of AI faces critical challenges, including ethical concerns such as data privacy, high computational costs, and resource allocation disparities. This article explores the current landscape of non-invasive CAD assessment, highlighting the transformative potential and associated challenges of AI integration.

## Introduction and background

Coronary artery disease (CAD) remains a leading cause of morbidity and mortality in the United States and globally, contributing significantly to socioeconomic burdens [1]. In 2022, there were 315 million prevalent cases of CAD worldwide, with an age-standardized prevalence of 3,605 (2,892-4,454) per 100,000, reflecting an 18% decrease from 4,390 per 100,000 in 1990 [2]. Notably, Central Europe, Eastern Europe, and Central Asia exhibited the highest age-standardized prevalence at 8,019 per 100,000, while South Asia recorded the lowest at 2,393 per 100,000 [2]. The reduction in CAD-related mortality in developed countries over recent years has been largely attributed to advancements in acute treatments and technological innovations that enhance both primary and secondary preventive strategies [3]. However, significant disparities persist across different ethnic and socioeconomic groups, impacting outcomes due to unequal access to effective treatment and preventive measures, even within the same country [4].

CAD is characterized by an inadequate supply of blood and oxygen to the myocardium, typically caused by coronary artery occlusion, leading to an oxygen demand-supply mismatch. The condition is often associated with plaque formation within the coronary arteries, restricting blood flow. The evaluation of CAD requires a comprehensive understanding of its etiologies, which are classified into modifiable and non-modifiable risk factors. Modifiable risk factors include lifestyle and dietary habits that can be corrected, such as poor diet, obesity, smoking, hypertension, and abnormal lipid levels [5]. Regional dietary habits also influence CAD risk, with a higher prevalence in Western countries due to the consumption of fast food and unhealthy meals, increasing the likelihood of dyslipidemia and CAD [5]. Smoking remains the most significant risk factor for CAD and is still prevalent in the United States, affecting 15.5% of the population [6]. Dyslipidemia, characterized by elevated low-density lipoprotein (LDL) and reduced high-density lipoprotein (HDL), further contributes to CAD risk. Obesity, particularly abdominal obesity measured by waist circumference, plays a critical role. Studies have shown that individuals with obesity have a higher CAD risk, but even those with a normal BMI and elevated waist circumference are similarly at increased risk [7,8]. Non-modifiable risk factors include age (with risk increasing with advancing age), gender (higher risk in males compared to females), family history, and genetic predisposition [5]. Genetic predisposition plays a crucial role in CAD risk, with genome-wide association studies identifying specific genetic variants, such as those in the 9p21 locus, that are strongly linked to increased susceptibility, independent of traditional risk factors [9].

Non-invasive imaging modalities are crucial for the evaluation and management of patients with known or suspected CAD [1]. These include echocardiography (ECHO), coronary computed tomographic angiography (CCTA), cardiac magnetic resonance (CMR), and single-photon emission computed tomography (SPECT) myocardial perfusion imaging [10]. Each modality serves a distinct purpose: ECHO is widely accessible and useful for functional assessment, CCTA provides high-resolution anatomical visualization of coronary arteries, CMR offers superior tissue characterization without radiation exposure, and SPECT evaluates myocardial perfusion to assess ischemia and viability. Their complementary use enhances diagnostic accuracy and guides clinical decision-making based on patient-specific needs. Recent technological advancements, particularly artificial intelligence (AI), have begun to transform CAD management. AI, which mimics human cognitive functions by processing and analyzing vast amounts of data, is increasingly integrated into medical practice to enhance diagnostic accuracy, detect abnormalities, and provide tailored treatment recommendations [11]. AI algorithms can support clinicians by analyzing imaging data, identifying subtle discrepancies, and offering a clearer understanding of disease progression [10].

This article provides a comprehensive review of non-invasive CAD evaluation methods and explores the integration of AI, highlighting its potential benefits, current applications, and challenges in clinical practice. References were primarily sourced from PubMed, Google Scholar, and reputable journals, spanning 2005 to 2025, and only peer-reviewed, English studies relevant to CAD imaging and AI were included.

## Review

To evaluate CAD, it is important to understand the factors and mechanisms involved in its development. 

Pathophysiology of CAD

The hallmark of CAD development is the formation of atherosclerotic plaque, characterized by the accumulation of fatty materials within the arterial lumen, leading to narrowing and obstruction of blood flow. The process begins with the formation of fatty streaks through the subendothelial deposition of lipid-laden macrophages, known as foam cells [3]. In response to vascular injury, the intimal layer of the vessel wall becomes compromised, allowing monocytes to migrate into the subendothelial space where they differentiate into macrophages and engulf oxidized LDL particles, forming foam cells. These vascular insults also trigger an inflammatory cascade with the release of cytokines and the activation of growth factors, which further promote the deposition of oxidized LDL and collagen and an increase in foam cells [3].

The progressive accumulation of these elements leads to the formation of subendothelial plaques. Over time, plaques may stabilize if no further endothelial injury occurs. However, recurrent risk factors, such as hypertension, hyperlipidemia, and smoking, as well as additional insults like inflammation and oxidative stress, can cause the lesion to become hemodynamically significant. As time passes, myocardial tissue perfusion may become insufficient, triggering angina symptoms during times of increased oxygen demand (e.g., exercise) if the lumen has at least 70% obstruction [3].

Importantly, certain plaques are prone to rupture, exposing tissue factors that trigger thrombosis. This thrombotic event can result in subtotal or total occlusion of the artery, leading to acute chest pain. Severe and acute obstructions often manifest as acute coronary syndrome (ACS), presenting clinically as unstable angina, non-ST-elevation myocardial infarction (NSTEMI), or ST-elevation myocardial infarction (STEMI), depending on the severity and extent of the ischemic insult [3,12]. In addition, coronary artery vasospasm can contribute to CAD by temporarily reducing blood flow, which may exacerbate symptoms, particularly in the context of underlying atherosclerosis [13].

Non-invasive evaluation of CAD

Although classical symptoms of CAD are well recognized, many individuals present with atypical symptoms that may not be detected through initial evaluation involving history-taking, physical examination, and a 12-lead ECG. These cases often require further imaging. Failure to pursue additional evaluations may result in a missed CAD diagnosis. Studies have shown that 2-5% of patients with myocardial infarction (MI) are mistakenly discharged from emergency departments (ED), leading to worse outcomes [14]. Several non-invasive diagnostic modalities, including ECG, echocardiography, chest radiography, stress tests, and serum markers, are essential for CAD assessment. The choice of diagnostic modality depends on the patient’s presentation.

Serum Markers

Blood tests are fundamental in the initial workup for CAD, providing key diagnostic and prognostic information. Cardiac enzymes, including high-sensitivity troponin and creatine kinase, are critical for identifying ischemic events. While biomarkers like troponin play a crucial role in CAD evaluation, their limitations, including false positives in conditions such as dehydration and sepsis, can complicate diagnosis and impact clinical decision-making, potentially leading to unnecessary testing, delayed treatment, or misclassification of risk. Inflammatory markers like C-reactive protein (CRP) and erythrocyte sedimentation rate (ESR) can point to inflammatory cardiac diseases, including pericarditis [3]. Liver function tests (LFTs) may indicate right-sided heart pressure elevations, termed congestive hepatopathy, or suggest infiltrative processes like hemochromatosis.

Electrocardiogram

The ECG is a cornerstone of CAD diagnosis, particularly in ACS. A 12-lead ECG captures the heart’s electrical activity, correlating each lead with specific cardiac regions [3]. ACS may manifest as ST-segment or T-wave abnormalities. Chronic cardiac conditions such as left axis deviation, bundle branch blocks, and left ventricular hypertrophy (indicative of long-standing hypertension) can also be detected. The importance of ECG screenings has led to prehospital ECG acquisition by emergency medical services (EMS), enabling earlier interventions. However, ECG has limitations, including reduced sensitivity in detecting non-ST-elevation events and subtle abnormalities in early CAD or prior infarcts, which can lead to missed diagnoses [15]. Despite these advances, clinicians must maintain high suspicion in patients with atypical symptoms-especially women, the elderly, and those with diabetes.

Echocardiography

Echocardiography, or cardiac ultrasound, plays a pivotal role in CAD assessment in both inpatient and outpatient settings. It can identify regional wall motion abnormalities indicative of ischemic heart disease while also evaluating valvular pathology, chamber size, and systolic function. A sudden decline in systolic function provides crucial diagnostic and prognostic information in CAD evaluation. Newer techniques, such as strain imaging - including global longitudinal strain (GLS) and early systolic lengthening (ESL) before shortening - can be measured at rest and may help predict obstructive CAD [16]. However, echocardiography has limitations, including operator dependency and challenges in patients with poor acoustic windows, such as those with obesity or lung disease, which can affect image quality and diagnostic accuracy [17,18].

Stress Testing

Stress tests simulate cardiac stress to provoke symptoms or findings suggestive of obstructive CAD. The test is typically stopped if chest pain, significant ECG changes, syncope, or other concerning symptoms develop. Continuous monitoring and serial ECGs are performed before, during, and after the test. Various stress test modalities accommodate patient comorbidities. The choice between exercise and pharmacological stress testing primarily depends on the patient's ability to exercise adequately and achieve a target heart rate, with pharmacological testing preferred for those unable to exercise or with specific contraindications [19]. For instance, pharmacological agents like dobutamine or adenosine are alternatives for patients unable to exercise, with contraindications tailored to conditions such as tachyarrhythmias or reactive airway disease. Exercise stress testing aims to reach 85% of the predicted maximal heart rate, terminating early if adverse events such as chest pain, hypotension, or new ventricular arrhythmias occur [3,20].

Coronary Artery Calcium (CAC) Score and Computed Tomography Angiography (CCTA)

CAC scoring is a valuable non-invasive modality, particularly for lower-risk patients for predicting CAD risk. A study involving 10,857 patients (2013-2020) demonstrated that a CAC score of 0 excluded obstructive CAD in 98.2% of cases and high-risk CAD in 99.4% [21]. Obstructive CAD is diagnosed on CCTA when one or more vessels show ≥50% stenosis, while high-risk CAD involves significant obstruction in the left main stem, proximal left anterior descending artery (LAD), or all three major coronary territories with proximal segment involvement [3]. These findings underscore the utility of CAC in initial evaluations for symptomatic but lower-risk patients [21]. However, cardiac CT examinations expose patients to ionizing radiation, which may pose potential health risks that require consideration [22]. Full CCTA may be warranted in patients younger than 45 years to definitively rule out CAD [21].

Integration of artificial intelligence and machine learning

The non-invasive assessment of cardiac structure and coronary vessels has undergone a transformative shift with the advent of AI and machine learning (ML). These technologies offer the promise of not only improving diagnostic accuracy but also providing additional predictive insights that are often too subtle for human eyes to detect [23]. The integration of AI and ML into the interpretation of imaging data has revolutionized the field, with AI algorithms now playing a central role in interpreting complex medical images, particularly in CAD. These advancements offer a much-needed solution to the challenges faced by clinicians in diagnosing CAD and predicting its progression. AI models like the deep learning (DL) algorithm described in Miller et al. [24] have been validated for myocardial perfusion imaging (MPI), demonstrating improved risk prediction for major adverse cardiovascular events (MACEs) by analyzing perfusion patterns and integrating clinical data. This model was trained and tested using a large cardiac imaging registry with internal cross-validation and external datasets, achieving impressive performance metrics such as high AUC values for event-specific risk predictions [24].

As we delve deeper into this evolving field, we will explore how AI and ML are being integrated into the non-invasive assessment of CAD and examine the potential impact of these innovations on cardiology’s future. Neural networks, the backbone of many AI applications, improve through a process known as experience, in which they analyze data and iteratively refine their models by constructing layered structures that enhance input-output processing [25]. This ability to learn from data allows neural networks to identify complex nonlinear relationships between input variables (such as clinical data or imaging features) and their resulting outcomes. By adjusting the weights associated with input data, the network reduces the overall error between predicted and actual outcomes, ultimately improving its predictive accuracy [25]. A more specialized subset of AI, DL, makes use of these neural networks to interpret large and complex datasets [26]. One particularly prominent neural network architecture used in medical imaging is the convolutional neural network (CNN), which has shown remarkable capabilities in automating feature extraction and classification from medical images. CNNs have played a key role in improving diagnosis and risk stratification in cardiology, helping physicians analyze imaging data with unprecedented speed and accuracy [27].

The use of DL algorithms allows clinicians to rapidly process large volumes of imaging data, including X-rays, CT scans, and MRI images, to identify subtle patterns and abnormalities that may not be readily visible to the human eye [28]. This ability to extract complex patterns from vast datasets enables the early detection of CAD and facilitates timely intervention. With the help of AI, algorithms can sift through cardiac images to identify small but significant changes in vascular structure, plaque buildup, and other indicators of heart disease. Moreover, AI has the potential to reduce unnecessary imaging studies by providing early and accurate assessments of cardiac health, allowing physicians to focus on the most pressing cases. In addition, these AI tools can assist physicians in adhering to evolving clinical practice guidelines, optimizing the use of imaging technologies to ensure that they are applied effectively and appropriately.

One of the most promising applications of DL in cardiology is in the analysis of coronary angiography, a standard technique for evaluating coronary artery stenosis. Coronary angiography provides valuable information about the presence and severity of blockages in the coronary arteries, but it is an invasive procedure that carries inherent risks for patients. AI models have been developed to analyze coronary angiography images and predict the likelihood of stenosis with high sensitivity and specificity, offering a non-invasive alternative to traditional methods [29]. DL algorithms have demonstrated strong agreement with expert readers and have shown performance comparable to invasive diagnostic procedures, such as intravascular ultrasound, in measuring plaque volume and luminal stenosis. AI-powered analysis of coronary CT angiography (CCTA) has also proven highly accurate, aligning with expert interpretations of CAD and enhancing the classification of stenosis based on the Coronary Artery Disease Reporting and Data System (CAD-RADS) [30,31]. These developments have allowed for better identification of CAD risk, with DL models capable of predicting future myocardial infarctions in patients experiencing stable chest pain [32].

The primary benefit of these AI tools lies not only in improving diagnostic accuracy but also in mitigating the risks associated with invasive procedures, improving patient safety and comfort. By reducing the need for invasive testing, AI models help minimize the potential for complications, thus offering a safer alternative for patients. Moreover, the use of explainable AI techniques - models that provide insights into the reasoning behind their predictions - has greatly improved the transparency and interpretability of these technologies. This transparency helps build trust among healthcare providers and patients alike, as clinicians can better understand the rationale behind AI-generated diagnoses and treatment recommendations [33]. In particular, CCTA has become an increasingly valuable non-invasive method for detecting CAD. However, it is known to overestimate stenosis severity when compared to invasive angiography, which can lead to unnecessary treatments. The lack of transparency in AI decision-making undermines trust, reduces accountability, and risks perpetuating biases, as opaque systems make it difficult to assess fairness or identify flawed logic [34]. The integration of AI into CCTA analysis has led to the development of more accurate models that can assess stenosis severity and, importantly, determine whether a blockage is hemodynamically significant when combined with fractional flow reserve (FFR) assessments [23].

Integrating both clinical and imaging data is essential for improving CAD risk prediction. While imaging alone provides valuable information, the addition of clinical data such as patient history, comorbidities, and laboratory findings allows AI algorithms to gain a more complete understanding of a patient’s overall risk. By synthesizing these diverse datasets, AI can identify patterns that might otherwise be missed, providing deeper insights into CAD’s pathophysiology and offering clinicians a more holistic view of the patient’s condition [35]. This combination of imaging and clinical data paves the way for more effective and personalized care. For example, by identifying high-risk patients at an earlier stage, AI allows clinicians to implement preventive measures, such as lifestyle changes, pharmacological interventions, and targeted therapies, before the onset of major cardiovascular events [36]. In doing so, AI not only improves patient outcomes by enabling earlier interventions but also emphasizes the transformative potential of AI in reshaping cardiovascular healthcare [37]. Examples of FDA-approved AI models integrated into EHRs include Cleerly's CAD Staging System and Ultromics' EchoGo Pro, which provide automated CAD assessment through standardized reporting interfaces. The shift toward proactive, preventative care marks a significant departure from the traditional model, underscoring the importance of early detection and intervention in improving long-term patient health.

As research into CAD pathophysiology continues to evolve, AI and ML models are becoming increasingly adept at enhancing the value of diagnostic images, whether they are analyzed in isolation or in conjunction with clinical data. These advancements are poised to redefine the standard of care in cardiology, improving not only disease detection and prognosis but also clinical decision-making. By enabling more precise and personalized treatment strategies, AI-powered diagnostics offer a promising future for combating CAD. As these technologies continue to refine predictive models and increase the accuracy of diagnoses, the era of precision medicine in cardiology is on the horizon [26].

Novel imaging biomarkers and multimodal approaches

Novel imaging biomarkers, which include anatomic, physiologic, biochemical, or molecular parameters detectable through advanced imaging methods, have emerged as powerful tools to enhance diagnostic precision in non-invasive assessments of CAD [38]. These biomarkers offer valuable insights into the presence, severity, and progression of CAD, supporting more effective decision-making in clinical practice. One significant advancement is the assessment of coronary microvascular dysfunction (MVD), which enables the early detection of microvascular impairment, a key factor in understanding the pathophysiology of CAD beyond the epicardial coronary arteries [39]. MVD plays a critical role in CAD progression, and its evaluation through non-invasive methods provides essential information for identifying patients at risk for adverse cardiovascular outcomes.

Another key advancement in CAD imaging is the use of inflammation imaging, which provides a window into the inflammatory processes driving atherosclerosis progression. Targeted imaging of vascular inflammation facilitates early intervention and personalized treatment strategies aimed at modifying the course of disease [40]. For instance, positron emission tomography (PET) imaging with the metabolic tracer [18F]FDG is widely used in clinical studies of atherosclerosis to quantify vascular inflammation. PET imaging has been shown to exhibit higher uptake in patients with symptomatic carotid plaques compared to the asymptomatic contralateral side and healthy arteries. In addition, [18F]FDG uptake has been validated as a surrogate marker for plaque macrophage content in both animal models and human patients, underscoring its role in risk stratification and prognosis [41]. However, its utility is limited by high physiological FDG uptake in the myocardium, making it challenging to differentiate pathological uptake from background activity.

Angioscopy, an intravascular imaging technique, offers a unique advantage by enabling direct visualization of the plaque surface and luminal thrombus [42]. This technique allows for the evaluation of plaque characteristics such as composition, vulnerability, and the presence of intraluminal thrombi, providing a more comprehensive understanding of CAD pathogenesis, and it is a promising research tool. A prospective study involving 157 patients found that patients with yellow plaques - typically characterized by thin caps, positiveremodelingg, and high lipid content - were at an increased risk of developing ACS [43]. This finding emphasizes the importance of advanced imaging techniques in identifying high-risk plaques and guiding preventive treatment strategies.

Multimodal imaging approaches, which integrate various imaging modalities, are also becoming critical for comprehensive CAD evaluation and risk stratification. One such modality, coronary artery calcium scoring (CACS), is a pivotal tool for CAD risk assessment, as it quantifies the amount of calcium present in the coronary arteries. Although CACS has limitations, such as its inability to distinguish non-calcified plaque components, which can lead to the overestimation of CAD severity, advancements in CT imaging have enhanced its diagnostic accuracy and ability to assess CAD severity [44,45]. Furthermore, the integration of CACS with other imaging modalities has shown promise in enhancing its diagnostic and prognostic capabilities. For example, combining CACS with functional imaging techniques like stress echocardiography or MPI enables a more comprehensive evaluation of CAD progression, improving the precision of treatment strategies [39].

In addition to CACS, existing tools such as the Framingham risk score and the atherosclerotic cardiovascular disease (ASCVD) risk score provide important metrics for assessing the 10-year risk of coronary heart disease (CHD) based on traditional risk factors, including age, sex, race, blood pressure, serum cholesterol levels, smoking history, and the presence of diabetes [46]. These risk calculators offer valuable insights into the likelihood of future cardiovascular events, although emerging research increasingly focuses on personalized medicine to tailor treatment strategies based on individual risk profiles.

Recent advancements have also focused on the integration of functional and anatomical data to improve diagnostic accuracy in CAD assessment. Perfusion reserve and FFR measurements, when combined with anatomical imaging, offer a more comprehensive evaluation of coronary artery physiology and disease severity. Co-registration of functional and anatomical data is a promising approach that enhances diagnostic precision by correlating perfusion abnormalities with underlying coronary anatomy [45].

MPI remains a cornerstone for assessing myocardial blood flow and detecting ischemia. Established techniques, such as stress echocardiography, single-photon emission computed tomography (SPECT) MPI, and PET MPI, are all widely used for evaluating myocardial perfusion and identifying exercise-induced ischemia. Each modality offers distinct advantages in terms of sensitivity and specificity. For instance, SPECT has demonstrated an 88% sensitivity and 76% specificity for detecting CAD compared to invasive coronary angiography [47]. The development of newer imaging technologies, such as dynamic CT MPI, further enhances diagnostic accuracy by enabling the direct measurement of myocardial perfusion while reducing radiation exposure [48]. These advancements offer clinicians valuable tools for assessing myocardial viability and optimizing patient management strategies.

Overall, the integration of multiple imaging modalities and AI-powered techniques provides a more comprehensive and accurate evaluation of CAD. AI improves image reconstruction, noise reduction, and feature extraction by leveraging deep learning to enhance image fidelity across modalities like MRI, CT, and PET, as seen in accelerated MRI scanning and optimized CT reconstruction techniques [49]. These innovations not only enhance our ability to diagnose CAD at earlier stages but also allow for more precise risk stratification, better-informed treatment decisions, and improved patient outcomes. As these technologies continue to evolve, the future of CAD management will increasingly rely on personalized, multimodal approaches that combine anatomical, functional, and molecular imaging to guide the best possible care for patients.

Future directions and emerging technologies

Future directions in the non-invasive assessment of CAD are being profoundly influenced by innovations in molecular imaging technologies. These advancements have introduced novel tools that identify biological characteristics of atherosclerotic plaques that traditional anatomic imaging techniques fail to capture, opening new frontiers for CAD diagnosis and management [50]. Among these advancements, PET has emerged as a pivotal technique for assessing disease activity in real time. PET enables the detection of specific biological processes, such as plaque inflammation and macrophage activity, which are integral to atherosclerotic progression. When combined with CT in a hybrid PET/CT scanner, this technology allows for a more comprehensive assessment by integrating both anatomical and molecular data [51]. PET and SPECT, particularly when coupled with specific molecular tracers, offer high sensitivity and enable quantitative measurements that provide valuable insights into plaque composition, inflammation, and the potential for rupture within coronary vessels [50].

Alongside PET and SPECT, cardiac magnetic resonance (CMR) imaging has seen extensive clinical use in evaluating cardiovascular morphology, ventricular function, myocardial perfusion, tissue characterization, and CAD. At a molecular level, CMR can visualize critical cellular processes such as macrophage activity, which plays a significant role in plaque formation and rupture. In animal models, gadolinium (Gd) particles have been used to enhance MRI contrast, enabling the visualization of atherosclerosis and demonstrating a direct relationship between MR enhancement and the presence of macrophages [52,53]. The hybridization of MR and PET imaging has generated significant interest in recent years. CMR and PET both demonstrate high diagnostic performance for detecting CAD, with CMR excelling in soft tissue characterization and stress perfusion imaging, while PET provides superior quantification of myocardial blood flow and flow reserve, making them complementary modalities for clinical decision-making [54]. This combination offers a patient-friendly alternative to hybrid PET/CT scanners, as it reduces the radiation dose required for PET imaging while still providing detailed molecular and anatomical data. However, one of the major challenges faced by MR/PET imaging is attenuation correction, although several studies have investigated the validation of newly developed MR-based attenuation correction methods against the well-established and clinically accepted CT-based attenuation correction [55]. This process adjusts PET data to account for signal attenuation caused by body tissues and the MR scanner components, which can introduce artifacts.

 Nevertheless, studies have shown that the effect of attenuation correction on cardiovascular imaging is minimal, as the coils in MR scanners have a low absorption cross-section and are positioned at a considerable distance from the cardiovascular tissue [55,56]. Despite their diagnostic advantages, the widespread adoption of PET/MRI and hybrid imaging modalities is limited by high costs, restricted availability, and the need for specialized expertise, compounded by challenges like MR-based attenuation correction and reduced patient throughput, which hinder routine clinical implementation.

Another key development in non-invasive CAD assessment is the integration of advanced image analysis techniques, such as DL and ML. These technologies are increasingly being applied to extract valuable insights from molecular imaging data, offering new opportunities for precise risk stratification based on molecular signatures [57]. By harnessing the power of these technologies, clinicians can gain a better understanding of plaque characteristics, the likelihood of rupture, and the risk of adverse cardiovascular events. The combination of molecular imaging with existing non-invasive imaging techniques promises to enhance diagnostic accuracy, improve patient risk stratification, and inform more personalized treatment strategies. By identifying high-risk patients earlier, clinicians can implement targeted interventions that could improve patient outcomes and reduce the burden of CAD on healthcare systems.

The future of CAD assessment is also being shaped by the evolution of wearable and portable imaging devices, which provide continuous, real-time monitoring of cardiovascular health. Technologies such as bioimpedance spectroscopy (BIS), photoplethysmography (PPG), and arterial tonometry are emerging in CAD diagnostics, with ongoing research evaluating their clinical utility. While not yet standard in routine practice, these modalities can detect abnormal hemodynamics, such as arterial stiffness and irregular pulse waveforms, which may be suggestive of ischemia and contribute to early risk assessment [58]. These emerging tools allow for the real-time collection of cardiovascular data, enabling early detection of changes in cardiovascular health that could signal the onset of CAD [59]. In addition, wearable electrocardiography (ECG) patches have enabled continuous monitoring of heart activity, facilitating early detection of arrhythmias and potentially preventing adverse cardiac events [60].

The integration of biosensors for biomarker detection, along with point-of-care testing (POCT) for cholesterol levels and inflammatory markers, has further enhanced diagnostic capabilities. These technologies allow for rapid, on-site testing that can help identify patients at risk for CAD, facilitating early intervention and personalized treatment strategies [61]. POCT enables healthcare providers to make informed decisions more quickly, while remote monitoring via telemedicine platforms allows for ongoing management and communication between patients and healthcare providers, particularly in underserved areas or where access to specialists is limited.

The convergence of AI with POCT data also holds significant promise. By analyzing data collected from wearable devices, biosensors, and POCT, AI algorithms can identify patterns and provide real-time diagnostic insights. This integration streamlines the diagnostic process, improves treatment optimization, and facilitates more effective management of CAD on a global scale. As these technologies evolve, they will not only enhance the accuracy and efficiency of CAD diagnostics but also pave the way for more personalized, proactive care strategies, ensuring better outcomes for patients worldwide.

Overall, the integration of molecular imaging technologies, advanced image analysis techniques, wearable devices, and AI-powered diagnostic tools represents a major leap forward in the non-invasive assessment of CAD. These advancements promise to reshape CAD management by improving early detection, risk assessment, and treatment strategies, ultimately leading to better patient outcomes and a reduction in the global burden of cardiovascular disease.

Clinical implications and research opportunities

Non-invasive techniques have become integral in guiding treatment decisions for CAD, offering valuable insights into the necessity of medical therapy versus revascularization procedures such as angioplasty or bypass surgery. CT is optimal for assessing plaque burden, offering detailed insights into plaque dimensions and composition [62], while PET remains the most validated method for detecting microvascular dysfunction through precise myocardial blood flow quantification [63]. For ischemia, CMR excels in stress perfusion imaging and fibrosis assessment. By providing real-time data on disease progression and treatment response, these techniques enable healthcare providers to make timely adjustments to therapeutic regimens. This capability is essential for optimizing treatment plans and ensuring that patients receive the most appropriate interventions at each stage of their disease [64]. Furthermore, the ability to personalize treatment plans based on non-invasive assessments enhances patient care by aligning interventions with the specific pathophysiological mechanisms underlying the disease. This individualized approach not only improves clinical outcomes but also increases patient satisfaction, as treatments are tailored to their unique needs and conditions [65].

Cost-effectiveness analyses underscore the importance of non-invasive approaches in healthcare decision-making, particularly in optimizing resource allocation. Multiple studies have demonstrated that CCTA is a cost-effective alternative to invasive coronary angiography, with one study reporting an average cost saving of $789 per patient when CCTA was performed prior to cardiac catheterization at a 50% prevalence of CAD [66]. By reducing the need for more invasive procedures, these techniques contribute to streamlined workflows and help avoid unnecessary interventions, thereby lowering healthcare costs while maintaining or improving the quality of care. Studies have shown that non-invasive imaging techniques can significantly reduce the financial burden associated with traditional diagnostic methods, such as invasive coronary angiography, without compromising diagnostic accuracy [67,68].

The current trends in the development of non-invasive methods for CAD assessment are discussed, with a focus on advanced imaging techniques enabled by CT and MRI. These emerging methods hold the potential for detecting early signs of CAD that may be missed by conventional approaches. In addition, the use of combined imaging modalities, such as hybrid PET/CT and PET/MRI, offers a more comprehensive evaluation of CAD by providing both anatomical and molecular information for enhanced accuracy. PET/CT allows for the rapid and precise fusion of functional and anatomical data, making it ideal for assessing ischemia and myocardial viability. By contrast, PET/MRI excels in providing superior soft-tissue contrast and reducing radiation exposure [56].

The incorporation of AI in image analysis is another exciting development that promises to revolutionize CAD diagnostics. AI algorithms are increasingly being used to analyze complex imaging data, identifying patterns and abnormalities that may be difficult for human observers to detect. AI-powered systems can also assist in risk prediction by evaluating large datasets to identify individuals at high risk of developing CAD, enabling earlier intervention and more proactive management. These AI-driven tools are already being employed to automate the interpretation of images, streamline workflow, and improve clinical decision-making, allowing healthcare providers to focus on delivering personalized care.

Despite these advancements, several challenges remain, particularly in addressing the limitations of current non-invasive methods. Radiation exposure from imaging techniques such as CT and SPECT, as well as the high costs associated with some of these modalities, remain significant concerns. Protocols like low-dose CT mitigate radiation risk, helping to reduce exposure while maintaining diagnostic quality. Research is ongoing to develop imaging methods that minimize radiation exposure without compromising diagnostic accuracy. In addition, efforts to reduce the costs of advanced imaging technologies and improve accessibility, especially in resource-limited settings, include the use of AI-based automation and telemedicine. AI enhances radiology workflows by reducing scan and reporting times, leading to faster diagnoses and lower operational costs [69], while teleradiology minimizes patient travel and hospital resource use, further contributing to cost savings.

Challenges of AI integration into non-invasive CAD evaluation and potential solutions

Data Quality and Availability

AI models require large, diverse, and high-quality datasets for effective training, but this remains one of the greatest challenges in healthcare, particularly in cardiovascular imaging. The scarcity of diverse datasets - especially regarding disease prevalence, as well as racial and gender representation - impedes the ability of ML methods to perform optimally. AI models, particularly those used for CAD detection, depend on being trained on a wide-ranging dataset that represents a diverse population. A lack of dataset diversity often leads to biased data, which can hinder performance and reduce the generalizability of CAD detection tools across different populations [1]. For example, a study on AI for MPI found that models trained without data augmentation overestimated CAD probability in women [70]. Similarly, an ECG-based deep learning model underperformed in Black patients compared to White patients for conditions like aortic stenosis [71], highlighting how biased and underrepresented datasets impair diagnostic accuracy for both women and racial minorities. In healthcare, the difficulty of acquiring such datasets is compounded by concerns related to patient confidentiality regulations. Strict privacy laws, such as the Health Insurance Portability and Accountability Act (HIPAA) in the United States and the General Data Protection Regulation (GDPR) in Europe, impose significant restrictions on accessing large datasets for AI training. These regulations limit the availability of comprehensive datasets, making it challenging for AI models to be trained on representative, real-world data. In addition, the underrepresentation of minority groups or specific geographical regions further biases models, potentially exacerbating health disparities.

Another challenge lies in obtaining sufficient input from clinicians to annotate training data, as AI model accuracy is heavily dependent on the quality of labeled data. Expert annotation, such as cardiologists marking regions of interest on imaging modalities like echocardiograms or MRI scans, is time-consuming, costly, and subject to inter-observer variability, which further limits data consistency and quality [2]. Furthermore, missing or incomplete clinical data within datasets is a common issue that undermines their utility for training AI models.

To address the concern of data sharing and availability, the promotion of collaborative data-sharing networks is crucial. Initiatives like the National Institutes of Health (NIH) Bridge2AI project help create comprehensive datasets that improve generalizability and reduce biases. In addition, continuous bias auditing and data balancing can help mitigate disparities in demographic representation and foster more diverse datasets.

Regulatory and Ethical Concerns

The integration of AI into healthcare, particularly in non-invasive CAD evaluation, raises complex regulatory and ethical issues that must be addressed to ensure patient safety - a core principle of public health. These concerns revolve around ensuring AI systems are safe, effective, unbiased, and respectful of patient privacy [3]. The FDA and EU MDR each have distinct regulatory approaches for medical devices. The FDA (US) focuses on device classification, categorizing devices into Classes I, II, and III, with increasing regulatory control depending on the classification. By contrast, the EU MDR emphasizes a lifecycle approach, incorporating stricter clinical evidence requirements and the use of unique device identification [72].

In the United States, AI-based medical devices and software must meet regulatory standards set by the Food and Drug Administration (FDA) before clinical use. Similarly, standardization issues in molecular imaging arise due to variations in tracer uptake between patients and institutions, influenced by factors such as differences in PET/CT hardware, reconstruction methods, patient preparation, and uptake time, which can lead to variability in quantitative metrics like standard uptake value and radiomic features [73]. However, the dynamic nature of AI algorithms, especially those utilizing continuous learning or adaptive features, presents challenges to regulatory bodies to keep pace with technological advancements [3].

Therefore, developers, users, and regulatory bodies all share the responsibility of ensuring adherence to ethical principles during the development, deployment, and ongoing evaluation of AI systems in healthcare. Some of the key challenges in regulation involve determining when algorithmic changes necessitate re-review, which can vary across jurisdictions. In addition, traditional regulatory frameworks are not equipped to handle the rapid iteration cycles of adaptive AI, highlighting the need for innovative solutions [74]. To address this challenge, continuous post-market surveillance can monitor AI performance, and adaptive regulatory frameworks, such as the FDA’s proposed “predetermined change control plan,” may help guide AI integration into healthcare [4].

Patient safety and clinical validation are also major ethical concerns. AI models used for CAD evaluation must demonstrate clinical safety and effectiveness prior to widespread adoption. For instance, false positives or false negatives in diagnostic algorithms could lead to unnecessary interventions or missed diagnoses. AI models developed to detect obstructive CAD might overpredict or underpredict significant stenosis due to variations in data quality or patient characteristics, which could affect the reliability of results. 

To address these concerns, AI models for non-invasive CAD detection are validated through various methods, including retrospective validation studies, which primarily rely on historically labeled data but may not fully reflect real-world performance [75]. While prospective validation studies are limited, they are crucial for evaluating clinical utility [75]. In addition, external validation is essential for assessing generalizability across diverse populations and healthcare settings, with multi-center trials enhancing model robustness by exposing algorithms to varied datasets and clinical practices [76]. These issues can be mitigated by using diverse, comprehensive datasets for training and conducting rigorous clinical trials and multicenter studies.

Moreover, the risk of AI models inheriting biases from unrepresentative datasets can lead to disparities in diagnostic performance across different demographic or socioeconomic groups. For example, AI models trained on less diverse datasets may underperform in women or minority groups, thereby exacerbating health disparities. Regular bias audits, fairness evaluations, and the use of diverse and representative datasets can help alleviate this concern [3].

Transparency and explainability are also critical ethical issues. AI models, particularly those based on DL algorithms, are often difficult to interpret, making it challenging for healthcare providers to understand how decisions are made. Differences in imaging protocols, interpretation criteria, and operator experience may affect reproducibility. This opacity in AI decision-making is a significant barrier to regulatory approval and clinical adoption. To address this, explainable AI and interpretable ML models should be developed to provide clear explanations of their decision-making processes, thereby fostering public trust and clinical acceptance [6].

Data privacy and security are fundamental concerns as AI models require large volumes of patient data for training and continuous learning. Balancing data availability for AI development with the need to protect patient confidentiality is a major challenge. Regulations such as the HIPAA and GDPR restrict the use, storage, and sharing of patient data, limiting the availability of comprehensive datasets. Federated learning, along with advanced encryption and de-identification techniques, can protect patient privacy while enabling AI models to be trained on decentralized data.

Accountability and legal liability also present challenges. In the event of an AI-related error in patient diagnosis or care, it is unclear who bears responsibility - the physician, the AI developer, or the institution deploying the system. As AI evolves in CAD diagnostics, malpractice laws will likely adapt to address shared liability, redefine standards of care, and manage the complexities of AI’s role in medical decisions. Establishing clear liability frameworks that delineate roles and responsibilities, while promoting shared decision-making models where AI assists rather than replaces human judgment, can help mitigate these concerns.

Ultimately, the successful integration of AI into non-invasive CAD evaluation must align with the four principles of ethics-beneficence, non-maleficence, autonomy, and justice [3].

Cost and Resource Allocations

While AI in non-invasive CAD evaluation offers significant clinical benefits, it also presents substantial costs and resource demands that could limit its widespread adoption, especially in low-resource areas. The development, implementation, and maintenance of AI systems require substantial investment in research, infrastructure, and skilled personnel, which can be prohibitively expensive, particularly in low-income countries or resource-constrained settings. The costs of acquiring large datasets, maintaining AI models, and conducting ongoing training and updates further exacerbate these financial burdens.

A potential solution to this issue is the creation of public-private partnerships (PPPs) that encourage collaboration between technology companies, academic institutions, and healthcare systems. These partnerships could play a crucial role by combining resources, expertise, and efficiencies from both sectors while fostering innovation under regulatory oversight. In practice, PPPs operate through shared responsibilities, where the public sector retains ownership and oversight to safeguard public interest; risk allocation, where risks are distributed based on each partner’s ability to manage them; and performance-based contracts, where private partners’ profits are tied to performance metrics, ensuring accountability and efficiency. By fostering these partnerships, AI integration in cardiovascular imaging can progress while addressing challenges related to funding, implementation, and equitable access. Additionally, global funding initiatives, such as those supported by the World Health Organization (WHO) or philanthropic entities, can help subsidize AI adoption in low-resource settings, reducing health disparities.

One approach to improve cost efficiency and resource allocation in resource-limited settings is by developing lightweight, cloud-based, transfer-learning AI models. These models, designed to operate with lower computational power, bandwidth, and data availability, can be tailored for specific applications like telemedicine, thereby promoting greater adoption and maximizing impact in these areas. Finally, raising awareness and fostering public acceptance of AI will be essential to facilitate its adoption and integration into healthcare systems, particularly in regions that stand to benefit the most from advanced diagnostic technologies.

## Conclusions

The non-invasive evaluation of CAD has significantly advanced, driven by innovations in imaging modalities and AI integration. Future advancements in CAD assessment will continue to be driven by imaging technologies such as PET, SPECT, MRI, and hybrid imaging, along with AI applications like DL for risk stratification and federated learning for secure data sharing. Ethical AI implementation, regulatory approval, and physician adoption will be critical in shaping the future of CAD diagnostics. Transparent and explainable AI models, alongside robust regulatory frameworks, are necessary for building clinical trust and facilitating integration into practice. However, the widespread adoption of these advancements remains limited by challenges related to data quality, diversity, accessibility, and cost, which must be addressed to unlock their full potential.
